# The effectiveness of kinesiology taping on balance, gait, and gross motor function in the lower limbs of children with cerebral palsy: a systematic review

**DOI:** 10.1590/1806-9282.20240300

**Published:** 2024-08-16

**Authors:** Seth Kwame Agyenkwa, Duaa Abualkhair, Rustem Mustafaoglu, Ahmet Abo Orabi

**Affiliations:** 1 Istanbul University- Cerrahpasa, Institute of Graduate Studies, Department of Physiotherapy and Rehabilitation – İstanbul, Turkey.; 2 Istanbul University- Cerrahpasa, Faculty of Health Sciences, Department of Physiotherapy and Rehabilitation, Division of Physiotherapy and Rehabilitation – İstanbul, Turkey.

## INTRODUCTION

Cerebral palsy (CP) encompasses a range of motor impairment disorders and is the most common cause of physical disabilities among children in high-income countries, with an incidence of 2.11 per 1000 births^1,2^. Lower extremity dysfunctions in children with CP affect crucial activities for mobility and daily functioning, including postural control, functional mobility, sit-to-stand transfers, and gait abnormalities^3^.

Rehabilitation for children with CP is aimed at enhancing gross motor function (GMF), postural control, functional mobility, and independence^4,5^. Physiotherapy interventions, including neurodevelopmental therapy, manual stretching, splints, adaptive furniture, and orthosis, are commonly used, but their effectiveness remains inconclusive^6–8^. Children with CP often have reduced sensory stimuli reception and sensory-motor integration deficits, indicating a need for rehabilitative techniques that stimulate sensory pathways and promote muscle activation, like kinesiology taping (KT)^9–11^.

Previous research primarily focused on the impact of KT on the upper limb rather than the lower extremity^6–8^. Clinical trials have shown the effects of KT on lower extremity functional outcomes, including improvements in sit-to-stand (STS) and timed up-and-go (TUG) tests, better performance in the lateral step-up test, and enhancements in functional independence, GMF, and balance^9,11–13^. However, inconsistencies exist in the literature, with some studies not reporting significant improvements after KT application^3,14^. The aim of this review is to determine the effects of KT application on lower limb functional outcomes in children with CP.

## METHODS

The study was conducted according to the criteria in the Preferred Reporting Items for Systematic Reviews and Meta-Analyses (PRISMA) Statement^15^. The study was registered in the PROSPERO database with the registration number CRD42023464972.

### Search strategy

The databases searched included PubMed, Web of Science, PEDro, and Cochrane, as well as a manual search in Google Scholar. Keywords including "Kinesio-tape OR K-tape OR taping," "Cerebral palsy," "Lower limb OR Lower extremity," "Function," and "Gait" were used by two authors independently. Studies published in English between January 2000 and September 2023 were searched, and citations were imported into Endnote for deduplication.

### Eligibility criteria

Only clinical trials that assessed the effects of KT on lower limb functional outcomes in children clinically diagnosed with CP were included in this review. Studies were excluded if subjects had undergone any orthopedic surgery or received a botulinum toxin injection in the 6 months preceding the evaluation date.

### Methodological quality

The Cochrane Risk of Bias (ROB) tool was used to assess studies according to random sequence generation, allocation concealment, blinding of participants and personnel, blinding of outcome assessors, intention to treat, and description of exclusion and losses^16^.

### Data extraction

Two authors independently screened all titles, abstracts, and full texts for eligibility. The disagreement over inclusion was resolved through a consensus meeting with a third reviewer. The relevant data from the included studies were extracted and presented in Table 1.

**Table 1:** Description of the characteristics of sample demographics, interventions, outcome measures, and results of the included studies.

Author	N (EG/CG)	Severity of CP	Age (years)	Treatment	Purpose/location of tape	Duration	Outcome measures	Results
Costa Brazil	4	GMFCS I and II	9–11	EG: KT CG: untreated	-Muscular activation -Ankles, hip, and sacral region	1 day	STS PBS TUG	Significant decrease in TUG, but not in STS and PBS (p>0.05).
Şimşek et al. Turkey	30 15/15	GMFCS III and IV	6.87	EG: KT+PT CG: PT	-Postural alignment -Paraspinal musculature	12 weeks	GMFM Wee-FIM	No significant improvements in GMFM and Wee-FIM (p>0.05) when compared to CG post-intervention.
Santos et al. Brazil	11	GMFCS I and II	6–12	EG: KT CG: Placebo	-Postural alignment -Rectus femoris muscle	1 day	STS	Decreased duration to perform STS in elevated sitting when compared to without taping (p=0.046) and placebo (p=0.044).
Kaya Kara et al. Turkey	30 15/15	GMFCS I and II	9.7	EG: KT + PT CG: PT	-Functional correction -Hip abductors and knee extensors	12 weeks	Wee-FIM BOTMP STS	Significant improvements in STS, BOTMP, Wee-FIM in the EG (p<0.05) when compared to CG.
Partoazar et al. Italy	38 19/19	Not specified	10.79	EG: KT CG: Sham	-Function and balance -Paravertebrals	2 days	BBS TUG	Significant increase in BBS and TUG in EG (p<0.001), no significant changes in CG.
Özmen et al. Turkey	19	GMFCS I and II	11.62	EG: KT	-Muscle activation -Gastrocnemius and tibialis	2 days	TUG PBS	Significant improvement in TUG and PBS after KT application (p<0.05).
Ghalwash et al. Egypt	14 7/7	GMFCS III	6.19	EG:KT+PT CG: Knee cage+PT	-Postural alignment and control -Posterior–anterior knee.	12 weeks	GMFM	There was no significant difference between the two groups post-treatment (p>0.05).
Tabatabaee et al., 2019 a Iran	30 15/15	GMFCS I–III	6.93	EG:KT+OT+PT CG: OT+PT	-To improve muscular activity -Ankle and tibialis muscle	14 days	BBS FFR	Day 2: no significant improvement in both EG and CG, Day 14: significant differences in BBS only in the EG (p<0.001).
Tabatabaee et al., 2019 b Iran	30 15/15	GMFCS I–III	6.93	EG: KT+PT CG: sham + PT	-Improve function -Anterior–posterior lower limb	14 days	TUG	Significant changes in functional mobility only in the EG (p<0.05).

BBS: Berg Balance Scale; BOTMP: The Bruininks-Oseretsky Test of Motor Proficiency-version; CG: control group; EG: experimental group; FFR: forward functional reach test; GMFCS: gross motor function classification system; GMFM: gross motor function measurement; KT: kinesiology taping; OT: occupational therapy; PBS: Pediatric Balance Scale; STS: sit-to-sStand; PT: physiotherapy; Wee-FIM: The Functional Independence Measure for Children; TUG: timed-up-and-go.

## RESULTS

**Figure 1:**
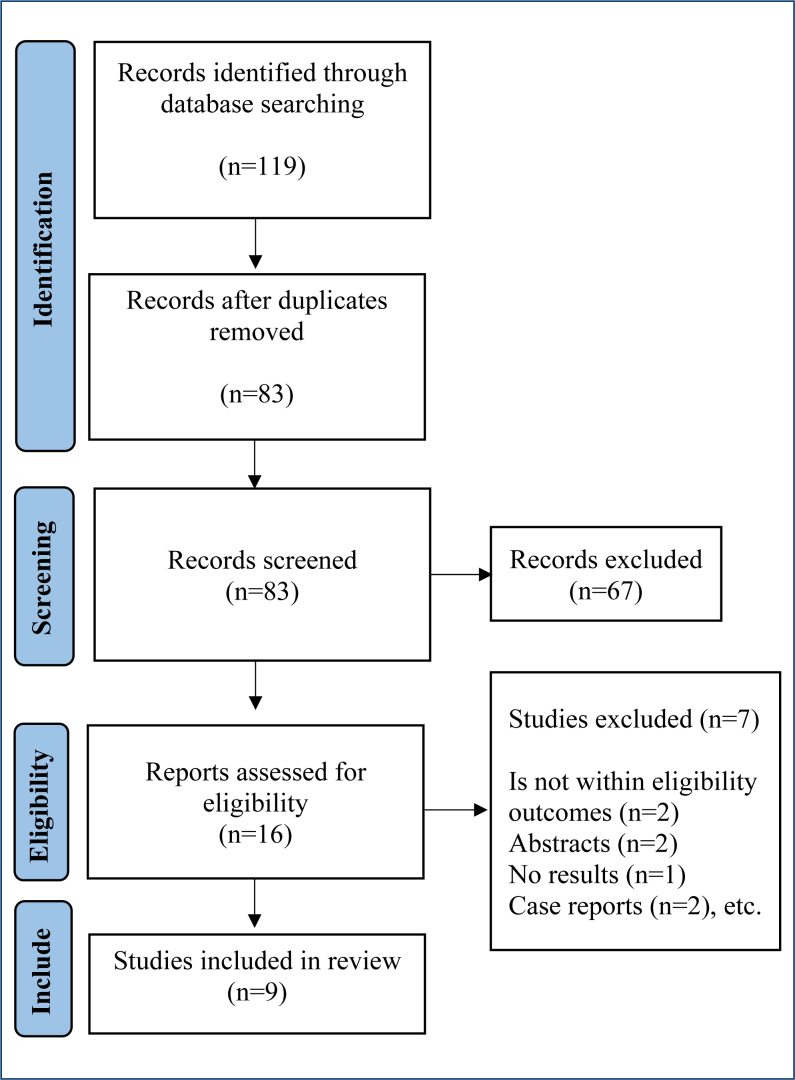
The PRISMA flowchart of the study selection procedure.

A total of 119 papers were retrieved from databases such as PubMed (n=11), Web of Science (n=85), PEDro (n=7), and Cochrane (n=4), and 12 studies were identified by hand searching. After removing duplicates, 83 studies were screened based on their titles and abstracts. Subsequently, 16 articles were examined thoroughly, and 7 of them were excluded owing to reasons depicted in Figure 1. Finally, nine studies were included in this review. The studies were published between 2011 and 2022. Six of the trials were randomized clinical trials^5,11,13,14,17,18^; two controlled trials^3,19^; and one placebo-controlled repeated measure^10^. The review involved a total of 206 participants, with ages between 2 and 18 years, the majority of whom suffered from spastic CP. Table 1 provides the details of the study characteristics.

### Quality of studies

The risk of bias among studies was assessed with the Cochrane ROB tool. Two out of the nine studies scored high in terms of random sequence allocation^3,19^. Allocation concealment was clearly observed in four studies^10,11,13,18^. Blinding of assessors was only possible in two studies^10,13^, while no single study was able to blind participants. The details of the individual ROB of the studies are demonstrated in Table 2.

**Table 2:** The Cochrane Risk of Bias (ROB) assessment scores of included studies.

Study	Random sequence generation	Allocation concealment	Blinding of participants and personnel	Blinding of outcome assessors	Intention to treat analysis	Description of exclusion and loses
Costa et al.^3^	High	High	High	High	Low	Low
Şimşek et al.^14^	Low	High	High	High	Unclear	Low
Santos et al.^10^	Low	Low	High	Low	Low	Low
Kaya Kara et al.^13^	Low	Low	High	Low	High	Low
Partoazar et al.^11^	Low	Low	High	High	Low	Low
Özmen et al.^19^	High	High	High	High	Low	Low
Ghalwash et al.^18^	Low	Low	High	High	Low	Low
Tabatabaee et al.^5^	Low	Unclear	High	High	Low	Low
Tabatabaee et al.^17^	Low	Unclear	High	High	Low	Low

### Outcome measures

Three studies examined the GMF of the lower limb using the D and E components of the GMFM, which assesses standing, walking, running, and jumping^13,14,18^. GMFM comprises 88 items scored on a four-point scale across five domains. Two of these studies investigated the long-term effects of KT over 12 weeks^14,18^, while one focused on short-term effects over 1 week^13^. One study reported improvement in both KT and control groups, but the difference was statistically insignificant for both GMFM D and E components^18^. Another study found no significant difference between the KT and control groups (p>0.05)^14^. Kaya Kara et al. observed short-term effects and also found no significant improvement between KT and control groups (p>0.05)^13^. The Bruininks-Oseretsky Test of Motor Proficiency (BOTMP) is another tool used to evaluate GMF, demonstrating high reliability^20^. One study utilizing BOTMP reported a significant difference in GMF between KT and control groups^13^. Thus, only one study among those that evaluated GMF in children with cerebral palsy found improvement between the experimental and control groups.

Four studies evaluated performance using the TUG test, which measures functional mobility, balance, gait, and fall risk^21^. Costa et al. found a significant difference in TUG times between the KT group and the control (p=0.048)^3^, with the KT group showing faster completion times. Partoazar et al. observed no immediate effects of KT on functional mobility (p=0.32)^11^. Özmen et al. reported significant changes in TUG readings 48 h post-KT treatment but not immediately after application (p>0.05)^19^. Tabatabaee, Cheraghifard, et al. found no significant difference between the first and second TUG assessments in the KT group but observed improvement between the first and third assessments (p=0.001)^5^.

The Functional Independence Measure for Children (Wee-FIM) assesses functional performance in self-care, mobility, and cognition^22^. One study initially found higher Wee-FIM scores in the control group compared to the KT group, but after 12 weeks, the KT group showed significant improvement in Wee-FIM scores, with a substantial difference between the KT and control groups^13^. Şimşek et al. observed significant post-intervention improvement in Wee-FIM scores in the KT group compared to their initial assessment, while no significant change was noted in the control group (p<0.05)^14^.

The Pediatric Balance Scale (PBS) evaluates functional skills like rising from a seated position and reaching beyond one's base of support^23^. Costa et al. found an increase in mean PBS-dynamic scores in the KT group compared to the control but no significant change in mean PBS-static scores (p=0.102)^3^. Two studies also examined balance using the Berg Balance Scale (BBS), with Partoazar et al. reporting a significant immediate rise in BBS scores post-KT application and removal^17^, while Tabatabaee, Shamsoddini, et al. found no short-term difference in BBS scores between KT and control groups but observed a significant long-term improvement in the KT group^17^. These findings suggest inconsistency in the effectiveness of KT in improving balance outcomes among children with cerebral palsy.

## DISCUSSION

The aim of the review was to determine the therapeutic effects of KT on the lower limb functional outcomes of children with CP. The review showed that KT does not enhance GMF in children with CP. Nonetheless, functional mobility could be significantly improved with KT when coupled with conventional PT. Application of KT targeting specific muscles of the trunk and lower limb may also improve balance outcomes.

The review found that KT did not enhance GMF, especially in severe cases. Although some studies showed improvements in specific measures like GMFM D and E and BOTMP scores when KT was used alongside conventional PT, overall, there were no significant differences compared to groups without KT or control groups^13,14,18^. Other reviews also support this, with only limited evidence suggesting improvements in GMF with KT application^6–8,24^. The short duration of the KT application may contribute to the lack of significant improvement in GMF, as the rehabilitation of children with CP typically progresses slowly. Overall, the data suggest that KT may enhance functional mobility in children with CP, particularly with consecutive applications over time. Partoazar et al. observed significant decreases in TUG duration over time in the KT group but not in the control group^11^. Another study found no significant difference in TUG scores between the KT group and a sham group after 2 days but noted a significant difference after 2 weeks of intervention^17^. Another study reported no immediate effects of KT on TUG, but significant improvements were seen after 2 days^19^. However, Costa et al. found significant improvement in TUG immediately after KT application^3^. Three of the four studies reported significant improvements in balance among children with CP who received KT^5,11,19^, while KT was therapeutically ineffective among children with CP in one study^3^. Balance is important to provide children with CP with the ability to achieve physical movement, perform basic activities of daily living, and participate safely in the environment.

Overall, there was an improvement in functional independence^13,14^, but only one study found a significant improvement in the group receiving KT compared to the group without KT^13^. With regards to leg strength and endurance, one study reported a decrease in the duration of STS immediately after KT application^10^, while another showed substantial improvement in STS after 12 weeks of KT application compared to physiotherapy only^13^. However, in a study that measured only the immediate effects of KT, there were peak values in STS without significant differences between baseline and final values, possibly due to the short duration of KT application^3^.

The studies reviewed aimed to improve muscle activation and activity in children with CP using KT. Despite similar goals, each study employed different KT methods, including specific taping techniques like Helen Hayes marker placement, Y banding, and I-banding. KT was utilized for various purposes, such as postural alignment, balance improvement, and reducing spasticity. Overall, the studies demonstrated consistency in therapeutic goals but utilized diverse approaches to KT application^3,5,10,13,19^. The studies used in this review may be at higher risk of bias due to the impracticality of blinding patients and researchers. Moreover, the small sample sizes limit the generalizability of the findings. The outcomes measured in these studies offer limited insight into the social integration and participation of children with CP after applying this modality. Future research should focus on developing feasible methods for blind participants and researchers to reduce bias and improve outcome measurement accuracy.

## CONCLUSION

The review shows that the KT application does not enhance gross motor gains when compared to conventional PT. However, functional mobility could be improved with KT application when coupled with conventional PT. Due to the slowness of functional recovery among children with CP, it is recommended to apply KT consecutively for at least 12 weeks.
